# Neuron-Glia Interactions in Neurodevelopmental Disorders

**DOI:** 10.3390/cells9102176

**Published:** 2020-09-27

**Authors:** Yoo Sung Kim, Juwon Choi, Bo-Eun Yoon

**Affiliations:** 1 Department of Molecular Biology, Dankook University, Cheonan 31116, Korea; ysungkim@dankook.ac.kr (Y.S.K.); 72201386@dankook.ac.kr (J.C.); 2 Department of Nanobiomedical science, Dankook University, Cheonan 31116, Korea

**Keywords:** neurodevelopmental disorder, neuron-glia interactions, ASD, ADHD, epilepsy

## Abstract

Recent studies have revealed synaptic dysfunction to be a hallmark of various psychiatric diseases, and that glial cells participate in synapse formation, development, and plasticity. Glial cells contribute to neuroinflammation and synaptic homeostasis, the latter being essential for maintaining the physiological function of the central nervous system (CNS). In particular, glial cells undergo gliotransmission and regulate neuronal activity in tripartite synapses via ion channels (gap junction hemichannel, volume regulated anion channel, and bestrophin-1), receptors (for neurotransmitters and cytokines), or transporters (GLT-1, GLAST, and GATs) that are expressed on glial cell membranes. In this review, we propose that dysfunction in neuron-glia interactions may contribute to the pathogenesis of neurodevelopmental disorders. Understanding the mechanisms of neuron-glia interaction for synapse formation and maturation will contribute to the development of novel therapeutic targets of neurodevelopmental disorders.

## 1. Introduction

Neurodevelopment occurs during the early stages of life. Maturation involves the consolidation of neural wiring, including synapse formation and synapse pruning [1]. Traditionally, synapse formation and pruning have been studied when investigating the generation and elimination of synapses. Synapses were thought of as the messenger for two adjusting neurons and that the elimination of synapses by pruning could establish synapse patterns that affect neuronal signaling [2,3]. However, recent studies have identified previously unknown features of glial cells that facilitate their interaction with neurons during brain development, such as the release of gliotransmitters and cytokines. In addition, synaptic changes induced by glial cells in the developmental stage, including synaptic patterning secondary to pruning, are now being investigated in relation to the neuron-glia interactions [4,5]. Glial cells release gliotransmitters such as gamma-aminobutyric acid (GABA), glutamate, and cytokines [6,7], which may affect neurons both directly and indirectly [8]. These substances are known to act at tripartite synapses [9].

**Figure 1:**
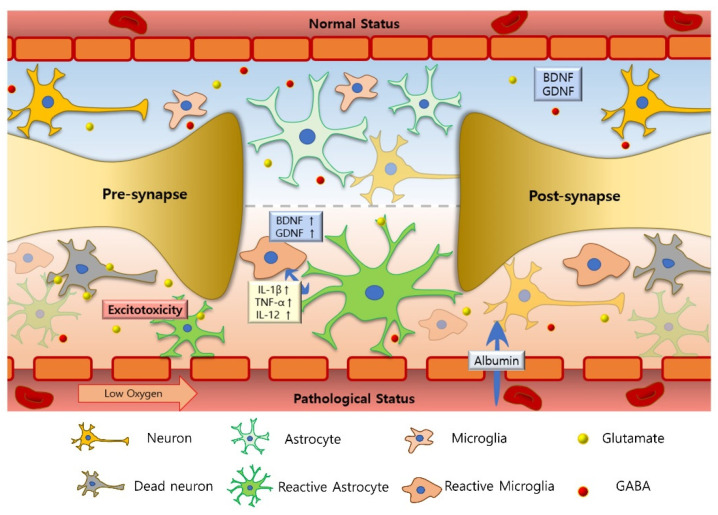
Changes associated with neuron-glia interactions in neurodevelopmental disorders. Inflammatory cytokines such as IL-12, IL-1β, and TNF-α are increased in neurodevelopmental disorders compared to normal individuals [13,14,15]. The increased cytokines activate astrocytes and microglia; these glial cells expand as a result of gliosis and release glutamate into the extracellular space, which kills the neurons. However, in patients with epilepsy, the blood-brain barrier opens, leading to the increased entry of albumin (↑) into the brain and astrocyte activation [16]. Unusually, in epilepsy, the released cytokines are known to facilitate functional neurogenesis and induce the release of neurotrophic factors such as neuronal growth factor (NGF), brain-derived neurotrophic factor (BDNF), and glial-cell derived neurotrophic factor (GDNF) (↑) [17,18,19,20].

**Figure 2:**
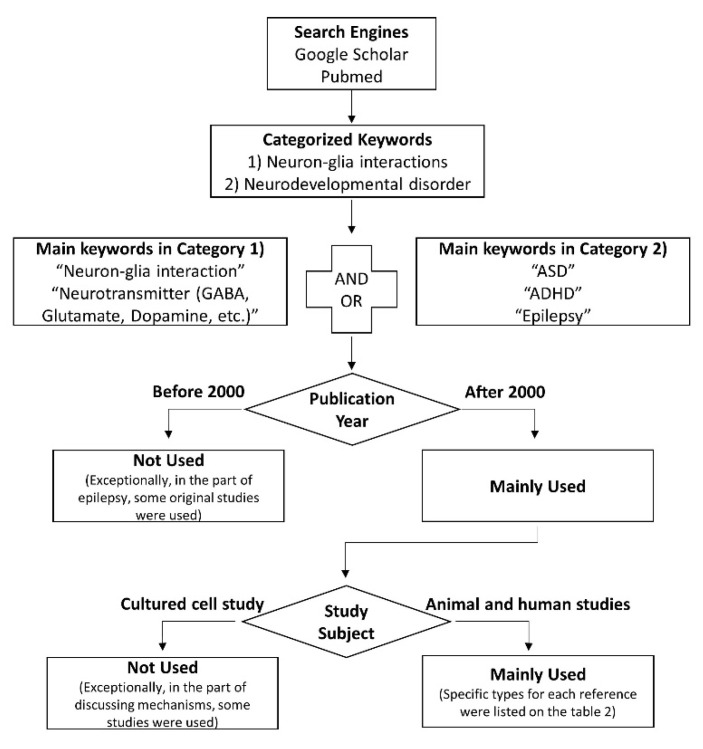
The search strategy used to identify references in this review.

Another important concept in neuron-glia interactions is the tonic release of gliotransmitters, typically manifesting with extracellular glutamate and GABA. Tonic inhibition has been invaluable for studying neurodegenerative disorders such as Alzheimer’s disease [10] and Parkinson’s disease [11]. However, this constant stimulation of nerve cells has rarely been studied in neurodevelopmental disorders [12]. In this review article, we introduce the concept of neuron-glia interactions as applied to several representative neurodevelopmental disorders, including autism spectrum disorder (ASD), attention-deficit/hyperactivity disorder (ADHD), and epilepsy (Figure 1). The search strategy used for the references identified in this review is presented in Figure 2 as a flow chart, and the characteristics of the included studies are shown in Table 1.

## 2. ASD (Autism Spectrum Disorder) and ADHD (Attention-Deficit/Hyperactivity Disorder)

ASD is a neurodevelopmental disorder that affects 1% of the population and there is currently no effective pharmacological treatment for ASD. There is a marked gender imbalance in ASD, with the rate being 4.3 times higher in the boys than in the girls [123]. For the majority of affected individuals, the cause of ASD remains unknown. ASD is characterized by a collection of neurobehavioral and neurological dysfunctions, including social and communication deficits, repetitive behaviors, and obsessive interests [21,179]. Previous studies have revealed that genetic and environmental factors contribute to autism. One of the environmental factors for ASD is maternal immune activation (MIA). Maternal viral infection, toxin exposure, and maternal obesity are linked to the dysfunction of inflammatory and immune pathways, which could increase the risk of behavioral deficits in offspring [124,125,151]. MIA is also known to be associated with ASD [99,100,126]. For example, mothers of children with ASD have been shown to have a higher frequency of allergies and autoimmune diseases compared to mothers with normally developing children [126]. In addition, the concentration of cytokines, including IFN-γ, IL-4, and IL-5, are reportedly elevated in the mid-gestation serum of females bearing a child with ASD [127,128]. Polyinosinic:polycytidylic acid (poly I:C) can mimic prenatal viral infection. MIA induced by the injection of poly I:C in a mother mouse model revealed that P2X_7_ purinergic receptors drive poly I:C-induced autism-like phenotypes such as social deficits and increased self-grooming [63]. This can induce an increase in inflammatory cytokines and impair neurogenesis, resulting in decreased Purkinje cell number and their density in the cerebellum as well as behavioral abnormalities in the fetal brain [101,157]. The decrease in GABAergic Purkinje cells in the cerebellum could cause a deficit in GABAergic transmission [101] and these neurodevelopmental dysfunctions may be related to ASD. In addition, MIA can alter the mammalian target of rapamycin (mTOR) pathway-associated genes, causing dysregulated inflammation [102]. MIA can be induced by poly I:C and prenatal virus infection [22].

On the other hand, it is known that environmental factors such as heavy metals can cause neurotoxicity. Lead (Pb) exposure disrupts Ca^2+^-dependent cell signaling and glutamatergic transmission though antagonization of the NMDA receptor [23]. Chronic methylmercury (MeHg) exposure directly inhibits Ca^2+^, glutamate, and GABA signaling [152]. Inhibition of the glutamine transporter in MeHg-exposed cultured astrocytes has been shown to result in a decrease in glutamate signaling [153]. In addition, GABA signaling decreased with modified GABA_A_ receptor conformation upon MeHg exposure [152].

There are numerous autism-related genetic factors, including genes such as *Shank3, neuroligin, neurexin, Pten, TSC1, MeCP2*, and *Scn1a* [64,65,66,158]. In particular, *Shank3, neuroligin,* and *neurexin* are widely known genes associated with ASD [24,25]; the other genes described above have been revealed by large-scale exome sequencing analyses [159,160]. Neuroligin and neurexin connect pre- and post-synaptic neurons at the synapse and mediate trans-synaptic signaling. Mutations in *neurexin* or *neuroligin* genes have been implicated in autism and other cognitive disorders [161,162] and may induce perturbations in synaptic transmission [67].

ADHD is one of the common neurodevelopmental disorders and contributes to children’s poor academic performance and deficits in social skills. Ten percent of school-aged children suffer from ADHD, and it is twice as common in males than in females [129]. The three traits that define ADHD are inattention, hyperactivity, and impulsivity [26,180]. Conventional treatment for children with ADHD, such as the neuroleptic drug methylphenidate, does not eliminate the root cause of the disorder and has some side effects, including sleep disorder [27]. Several animal models and human studies have attempted to elucidate the pathophysiologic mechanisms of ADHD. Recently, genome-wide association study (GWAS) and single nucleotide polymorphism (SNP) studies have identified ADHD-associated genes such as *GIT1* [68,149]. In addition to genetics, some studies have suggested other possible causes and risk factors, including exposure to environmental materials during pregnancy or at a younger age and the use of alcohol and tobacco during pregnancy [163,164].

Glial cells comprise 70% of all the brain cells [28] and help maintain a healthy central nervous system (CNS) environment through its role in synaptic maturation and plasticity. Therefore, understanding how glia affects neuronal activity is essential.

### 2.1. Neuroinflammation in ASD and ADHD

Recent research efforts have focused on neuron-glia interactions in the CNS, including dysregulated immune activities in various types of glial cells as well as the changes between resting-state and reactive glial cells. Microglia are the resident immune cells of the CNS and act as primary mediators of neuroinflammation [29]. Astrocytes perform similar functions as microglia in terms of immunity [30]. Microglia in healthy CNS tissue have a ramified shape in the resting state. When microglia are activated, they become ameboid in association with the phagocytosis of cellular debris, antigens, and synapse pruning. In the brain tissue of patients with ASD, the activated form of microglia have been observed [181]. Glial cells are known to secrete neurotrophic factors such as anti-inflammatory cytokines (IL-4 and IL-10), neuronal growth factor (NGF), brain-derived neurotrophic factor (BDNF), and neurotrophin-4/5 in the resting state [17,18]. In addition, microglia perform neuroprotective functions that contribute to neurogenesis and synaptic homeostasis [31]. Glial cells change their form when activated, responding to antigens associated with a variety of pathological states. Activated glial cells secrete proinflammatory cytokines such as IL-1β, IL-6, and TNF-α. However, cytokines derived from microglia could be cytotoxic to neurons and other glial cells, especially oligodendrocytes [13]. Excessive activation of glial cells drives systemic inflammation in the brain, which might cause synaptic elimination and dysfunctional synaptic plasticity [14]. Proinflammatory cytokines trigger the induction of excitotoxicity via an increase in glutamate release, alteration of ion channel expression, and immune activation of the blood-brain barrier. It has been suggested that an increased level of cytokines circulating in the blood due to immune activation could cause neurodevelopmental disorders in children during the neonatal period [130]. To confirm the effect of immune activation, more studies in children with ASD are required, especially considering that humans are subject to marked variations in the environmental and genetic factors. Cytokines secreted by glia affect various functions of the brain. For example, IL-1β is primarily a proinflammatory cytokine that increases in response to infection. This activates the innate immune system and induces the activation of immune cells such as T lymphocytes, B lymphocytes, and microglia. This dysregulation of IL-1β in the early stages of life should be considered when studying the mechanisms of neurodevelopmental problems, which include learning and memory deficits [69]. Highly elevated levels of IL-1β expression affect neural progenitor cell proliferation in the CNS and contribute to region-specific deviant brain growth in patients with ASD [131]. Neuropeptide neurotensin (NT) stimulates microglia to secrete IL-1β, which is increased in the cell cultures of human microglia obtained from children with ASD [103,132]. IL-1β increases the expression level of IL-6 and TNF-α, which are other proinflammatory cytokines contributing to the induction of apoptosis of neuronal cells [165]. IL-6 can modulate autism-like behaviors through impairments of synapse formation, dendritic spine development, and neuronal circuit balance [103,133]. However, IL-6 also has anti-inflammatory cytokine roles; this implies that IL-6 can participate in regulating metabolism and neuronal processes. Therefore, elevated IL-6 levels should be studied to evaluate its effect on the immune system.

TNF-α is secreted in a soluble/membrane-bound form and is known to induce apoptosis. In addition, microglia secrete chemokines, including CX3CR1 and CCL2. A function of chemokines is to recruit the immune cells. In the brain, macrophages secrete chemokines to attract microglia for phagocytosis. They release chemokines such as CX3CR1, CCL2, and CCL21, and they act as paracrine factors [166].

In addition, reactive microglia and astrocytes are known to produce reactive oxygen species (ROS). ROS are highly cytotoxic and lead to oxidative stress in neurons. Oxidative stress induces apoptosis; thus, chronic inflammation of the CNS may contribute to neuronal death by apoptosis triggered by ROS and inflammatory cytokines [32].

The abnormal cytokine profile of ASD patients suggests that immune system disturbance and abnormal neuroinflammation could be biomarkers of autism. These inflammatory changes in autism have been applied to rodent modes of ASD (Table 2).

Prenatal exposure to the antiepileptic drug valproic acid (VPA) has been reported to result in a high incidence of autism. Lucchina and Depino used a VPA-exposed ASD mouse model to study altered inflammation [71]. VPA-exposed mice showed reduced social interaction and increased depression-like behavior. In addition, long-lasting glial activation was observed in the hippocampus and cerebellum in association with lipopolysaccharide (LPS) stimulation. This observed activation of glial cells could help to explain the mechanism causing altered levels of cytokines in ASD [106].

Neuroinflammation in ADHD is a significant factor that can alter glial cell activity. This is supported by the finding that serum IL-6 levels in children with ADHD are increased compared to healthy children [134]. This implies that ADHD patients have dysregulated inflammatory responses [134]. Despite being an animal model of hypertension, the spontaneous hypertensive rat (SHR) has been used as a model of ADHD. The SHR model exhibits increased levels of inflammatory cytokines (IL-1b, IL-6, and TNF-α) in the serum, but decreased levels of TGF-β level. This model is limited by the presence of hypertension. However, the results of this rat model coincide with those of other studies on other older rodent models, used well earlier than the 1990s [73]. This highlights the need for a more suitable ADHD animal model and that research should be conducted in relation to the mechanism of ADHD.

An increase in oxidative stress was identified in a meta-analysis of patients with ADHD. The authors suggested that patients with ADHD had normal levels of antioxidant production, but the response to oxidative stress was insufficient [150].

On the other hand, it has been reported that methylphenidate hydrochloride (MP)-treated rats showed increased microglial activity in multiple regions of the brain (such as the insular cortex, hippocampus, and thalamus) [104]. MP is widely used in therapies for various psychiatric diseases, including ADHD and chronic sleep disorders. However, chronic MP treatment could mimic the pharmacokinetic profile of treated ADHD patients due to the blockage of the dopamine transporter (DAT) and norepinephrine transporter (NET) and the increased monoamine levels in the synapse. Chronic MP treatment affects the number of dopamine neurons in the substantia nigra (SN) as well as microgliosis. The mRNA expression of proinflammatory cytokine genes (IL-6 and TNF-α) is also increased in the acute MP-treated mouse brain [105]. Therefore, MP treatment is relevant to brain inflammation in ADHD.

### 2.2. Impaired E/I Balance in Patients with ASD and ADHD

One of the characteristic changes in ASD and ADHD is the E/I imbalance. Notably, increased glutamatergic excitation or reduced GABAergic inhibition occur in association with neuropsychiatric disorders including ASD, ADHD, and epilepsy. Previous studies have reported that astrocyte dysfunction associated with impaired glial glutamate uptake is a biomarker of numerous neuropsychiatric disorders [74]. Serum glutamate levels were elevated in children with ASD compared to controls. This serum glutamate change reflects altered glutamatergic neurotransmission [135]. Astrocytes are important in excitatory regulation to control extracellular glutamate levels via the glutamate transporters GLAST and GLT-1; in humans, these proteins are known as EAAT1 and EAAT2, respectively [34]. Various factors, including inflammatory cytokines, ROS, and genetic factors, affect the expression and function of glutamate transporters in astrocytes. GLT-1 knockouts have revealed astroglial dysfunction is associated with glutamate signaling. The study further showed an increase in repetitive behaviors and seizure severity in GLT-1 inducible knockout (iKO) mice. EPSC amplitude was also increased in GLT-1 iKO mice. GLAST affects locomotor hyperactivity and is exaggerated by the NMDAR antagonist, MK-801. This implies that the glial glutamate uptake is a deficit related to the pathophysiological risk factors for disease. This further suggests that GLAST KO contributes to hyperactivity related to psychiatric disorders [75].

Neuronal glutamatergic release and uptake are the principal signaling mechanisms in glutamatergic transmission. Astrocytes also participate in this signaling, and they uptake surplus glutamate that remains in the extracellular space to prevent the accumulation of glutamate and excitotoxicity. Generally, excitotoxicity is related to the activation of NMDARs by excessive glutamate, and brain cell death usually occurs after stimulation of glutamate receptors due to impaired reuptake of glutamate through GLT-1 transporters on glial cells [136]. It has been reported that the expression of these transporters is regulated by NF-κB [76]. One study in rats with upregulated GLT-1 expression, due to ceftriaxone, revealed that it severely impairs long-term depression (LTD) in mossy fibers of the rat hippocampal CA3 region. In addition, they revealed that chronic treatment with ceftriaxone altered long-term potentiation (LTP), which is associated with an increase in glutamate release [77].

Microglia also participate in glutamate signaling via the Xc- system. The Xc- transporter is a chloride-dependent antiporter that carries glutamate out of the cell and transports cysteine/cystine in at a ratio of 1:1 [35,78]. ROS are produced by the reactive form of microglia. ROS induce glutathione (GSH) shortages and initiate TLR4 signaling. TLR4 triggers elevated Xc- expression levels, resulting in cysteine/cystine influx and glutamate efflux [107]. ROS, TNF-α, and IL-1ß secreted from microglia impair EAAT function and increase extracellular glutamate levels. Taken together, reactive microglia interfere with neurotransmission by releasing excitotoxins such as glutamate, D-serin, adenosine triphosphate (ATP), and impaired glutamate uptake, and alter gliotransmitter release from astrocytes.

GABA is a representative inhibitory neurotransmitter that regulates the overall functions controlled by the brain, including learning and memory function [36,37]. Dysfunction in GABA transmission may be the pathological evidence of an E/I imbalance [38]. Astrocytes express GABA receptors (GABAR)—specifically, ionotropic GABA_A_ and metabotropic GABA_B_ receptors—and GABA transporters (GATs), including the GAT-1 and GAT-3 [79]. Through these receptors and transporters, glial cells actively interact with interneurons in a variety of brain regions [37]. In addition, astrocytes can regulate excitatory transmission by releasing ATP, which inhibits the activation of presynaptic adenosine receptors [80]. Furthermore, astrocytes synthesize GABA via monoamine oxidase B (MAOB) from putrescine in a different pathway to neurons [81]. The astrocytic bestrophin-1 (Best1) channel has been reported to mediate tonic inhibition by releasing GABA [12]. Therefore, astrocytes are crucial for regulating the E/I balance in the tripartite synapse. Additionally, reactive astrocytes can alter the expression level of membrane proteins and the activity of enzymes, which affect the tonic GABA levels. Previous studies reported a decrease in GABAergic interneurons and transmission in the ASD mouse model [82].

Presynaptic GAD1/2 is expressed in GABAergic neurons. Chao et al. studied an X-linked methyl-CpG-binding protein (MeCP2) mutation in a mouse model of ASD. They targeted the vesicular inhibitory amino acid transporter (Viaat, also known as Slc32al) to specifically delete the MeCP2 molecule in GABAergic neurons; therefore, GABAergic neuron-specific mutations in the Viaat-Mecp2^-/y^ mice down-regulated MeCP2 levels in GABA-positive neurons. These mice showed increased autism-like behavior such as repetitive behaviors. In addition, reduced Gad1/2 levels have been linked with decreased miniature IPSC (mIPSC) amplitude and electroencephalography-measured hyperexcitability [65]. Another study used the BTBR T+tf/J mouse strain as an ASD model to analyze the impairment of inhibition. BTBR mice had decreased GAD65 expression in the insular cortex, which is related to communication and social behavior. BTBR mice also showed impaired mIPSC, which implies weak inhibition in the insular cortex. Besides, other ASD model mice (Shank3 KO, MeCP2 KO) showed decreased GAD65 and parvalbumin expression levels. Weakened inhibitory transmission may affect E/I balance deficits and result in autism-like behaviors in ASD [70]. It has been reported that the deletion of Nav1.1 voltage-dependent sodium channel in ASD mice (Scn1a^+/−^) results in impaired GABAergic transmission due to a deficit in the firing of GABAergic interneurons. The benzodiazepine clonazepam, which is a positive allosteric modulator for the GABA_A_ receptor, recovered the behavior and GABAergic transmission [66].

Changes in glutamate and GABA levels in ADHD have also been investigated [39]. Changes in GABA levels have been reported in various regions, such as the cortex, striatum, and anterior cingulate cortex, and are an important trait of ADHD. Several studies have suggested that the decrease of GABA levels seen in ADHD patients might be related to the deficit of inhibitory actions in behaviors [137,138,167]. These decreased levels of GABA might be due to decreased glial GABA levels in the same region. The hippocampus and cerebellum have been reported to have reduced glial GABA levels, which might cause inattention and hyperactivity behaviors in animal models [83,84]. The GAT-1 KO mice exhibit hyperactivity, lack of attention, and learning deficiency [85].

However, other studies have reported no change or an increase in GABA levels. In children with ADHD, the subcortical GABA level was no different, while the GABA level in the subcortical and frontal regions in adults with ADHD was increased [182]. In addition, GABA release from striatal MSN neurons, by binding to the astrocytic GABA_B_ receptors, activates neuronal spiking and contributes to the hyperactivity with disrupted attention [86]. In a study using cadherin 13 (Cdh13) KO mice, the GABA levels remained unaltered in the hippocampal CA1 region. However, the mIPSC increased in the KO mouse [72]. In addition, the glutamate level was altered in the ADHD patients, and the gene expression levels related to glutamate showed significant differences compared to the controls [168]. Regarding the increase and decrease in GABA or glutamate, it is suggested that relative changes and balance are more important than absolute concentrations. Animal models may have different trends depending on their molecular and genetic characteristics, and human studies differ depending on the stage of the person’s studies life when the studies were conducted. Further animal and human studies are required to investigate more specific approaches as different aspects of both types of study can occur depending on the region and circuit of the brain studied.

## 3. Epilepsy

Epilepsy has been studied for a long time and the neurophysiologic traits of epilepsy are well-known, namely the abnormal heterogeneous and dynamic neuronal spiking resulting in ictal events in the brain [93,97]. Despite extensive studies on epilepsy, there have been relatively few studies on the effect of glial cells. Recently, several studies have been conducted to determine the function of glial cells in the epileptic brain. In addition, a review article was published to help elucidate trends in epilepsy studies [40]. The newly understood glial cell function of releasing gliotransmitters, including glutamate and GABA, changing the synaptic environment, and inducing the release of cytokines have allowed epilepsy to focus more on the neuron and glial interactions [41,87,154].

Conventionally, the main reason for epilepsy has been thought to be an increase in neuron-oriented glutamate and a decrease in GABA levels [42,43,88]. However, some studies have reported that several functions of glia contribute to the pathogenesis of epilepsy [44,45]. Epileptogenic cues activate microglia and astrocytes. These activated glial cells can increase the synthesis and release of proinflammatory molecules. Thus, glial cells contribute to neuroinflammation [46].

### 3.1. Neuroinflammation in Epilepsy

Neuroinflammation is caused by the release of cytokines from a variety of sources in the brain. Epilepsy is a common symptom in patients with brain tumors, head injuries, and genetic diseases [89,94,139,140]. Epileptic patients have been reported to have high levels of IL-1ß, IL-1 receptors (IL-1Rs), IL-8, IL-12, MIP-1b, translocator protein (TSPO), toll-like receptor-4 (TLR4), high mobility group box 1 (HMGB1), TNF-α, and cytokine-related genes [15,47,89,139,140]. IL-1ß and TNF-α are the most widely studied, and the concentration of these cytokines is known to be important for protogenic status and inhibition of seizures [48]. Rodent knock-out of laforin, encoding for the gene *Epm2a*, and malin, encoding for the gene *Epm2b*, exhibit seizures that are genetically induced. In this Lafora disease (LD) rodent model, upregulated inflammatory-related genes have been reported through RNA-sequencing (Table 3). Approximately 60% of the upregulated proteins were found to be related to microglia [89]. LD is a genetically induced epilepsy. Laforin and malin levels are decreased when genetically mutated, and polyglucosan bodies (PGBs) accumulate in the brain. PGB has been studied in neurons for a long time, but the fact that astrocytes also have a considerable portion of PGBs is a recent discovery. Astrocytic PGBs are significantly increased in malin KO mice, indicating that astrocytic PGB could also cause LD [90,169]. Therefore, further studies on neuron-glia interactions and pathogenesis are required using this genetic model of epilepsy.

Increased levels of inflammatory cytokines could act as a neuromodulator in the brain between glial cells and neurons [49,170]. Cell survival rates following cytokine release in epilepsy are highly dependent on concentrations [48]. Cytokines released from astrocytes reduce the survival rate of neurons by free nitric oxide (NO) and excitotoxicity to neurons, as described below [48,141]. Studies have also explored several treatments and recovery tests on glial cells in epilepsy. Treatment with vitamin E (α-tocopherol), miR-146a, and aucubin (AU) has been reported to reduce epileptic events [94,110,111]. Glial cell changes could reduce the inflammatory signals in the brain, and these reduced signals could stop stimulating the neurons around the glial cells. The α-tocopherol mentioned above could reduce IL-1ß and TNF-α levels by treatment in epileptic mice [112].

Compared to the control group, patients with epilepsies showed a several-fold increase in glial cells [111]. Activation of microglia and astrocytes is crucial to neurons and other types of cells that exist in proximity to glial cells [171]. Moreover, with the increase in inflammatory cytokines, chronically or overly activated glial cells are also frequently reported in epilepsy [111,142,143,155]. Chronic immune activation or the overexpression of TNF-α in glial cells can cause a synapse change that results in networks forming seizure-like activity patterns [50,91,98,113]. These reactive glial cells or gliosis imply that the recovery of glial cell function could reduce seizure events in epilepsy. In a post-traumatic epileptic rat model, increased glial reactivity was associated with the hyperexcitability of the neocortex region [155]. These activated glial cells have some loss-of-function changes. By recovering these glial cells, the frequency or amplitude of epileptic seizures could be rescued, which highlights the importance of glial cells in epileptic events. Through treatment with the antiserum of P2X_7_, an ionotropic receptor responding to extracellular ATP, seizure activity can be attenuated [114]. However, cytokines released from glial cells are not just neurotoxic. In the presence of IL-1β and TNF-α, astrocytes release NGF, BDNF, and glial-cell derived neurotrophic factor (GDNF) [51], which could explain why cytokines released in the brain cause neurodegeneration and neurogenesis in epilepsy [19,52].

Blood is another source that could introduce cytokines into the brain. Blood vessels are the main medium of all nutrients, oxygen, and cytokines. The change in permeability of the blood vessels changes the brain environment dynamically. Astrocytes are located close to blood vessels. The endfeet of astrocytes surround the vessels and contribute to neurovascular coupling (NVC) [20]. Astrocytic calcium levels are increased in the activated form. This astrocytic calcium signaling at the endfeet is related to ictal signals by the manipulation of vasoconstriction [20]. The endfeet of astrocytes are known to dilate the blood vessels at low oxygen levels [53]. In the biological model, low-oxygen levels resulted in increased epilepsy-like spiking patterns [41]. BBB dysfunction changes the permeability of molecules in the brain, and albumin flows into the hippocampus. Excessive albumin input changes the microenvironment and activates astrocytes [16,54]. The severity of BBB permeability dysfunction is positively correlated with seizure frequency [16].

Taken together, released cytokines following the activation of glial cells act on both neurodegeneration and neurogenesis in the epileptic brain. The dysfunction of the BBB could activate the release of these cytokines.

### 3.2. Impaired E/I Balance in Epilepsy

An increase in glutamate and a decrease in GABA levels was traditionally thought to be the main cause of epilepsy. In the AP-1 mouse model, the expression of GLAST is decreased [108]. In addition, the increase in glutamate levels could induce excitotoxicity and thus cause neuronal death [55]. Chronic epilepsy patients show significantly decreased numbers of neurons, even though the amount of neuronal loss is not known to be related to the type of seizure [144]. These excessive amounts of glutamate are buffered by glial cells in the brain [56,95]. In the sclerotic hippocampus of patients with mesial temporal lobe epilepsy (MTLE-HS), glial cells lose the glutamate metabolizing enzyme, glutamine synthetase [115]. The loss of astrocyte gap junction coupling is observed in human MTLE-HS [145].

Therefore, the main concept of epilepsy therapy relates to increasing the level of GABA or decreasing the level of glutamate. The tonic GABA could also reduce the severity of epilepsy in mice [12]. Changes in the GABA_A_ receptor subunits have been observed in a pilocarpine-injected epileptic mouse model; this change in the GABA_A_ receptor impaired both the phasic and tonic inhibitions [115]. GABA in astrocytes can be released via the Best1 channel and GAT [76]. Astrocytes can mediate approximately 75% of tonic inhibition by releasing GABA [116]. In epileptic mice induced using Best1 KO, the neural firing of the hippocampus was inhibited. Therefore, it has been suggested that astrocyte-released GABA could prevent seizure susceptibility [116]. This mechanism has the potential to be used as an anticonvulsant, as treatment with inhibitors of GABA uptake such as nipecotic acid relieve epilepsy in the audiogenic model DBA/2 [117]. Treatment with the glial GABA uptake selective inhibitor, THPO, delays the onset of epilepsy [109]. Thus, regulating glial GABA levels could be a useful target for epilepsy drug candidates and requires further studies [116]. Other treatments also alter GABA, releasing proteins in glial cells and thus suppressing neuronal activity. The expression level of GLAST is increased through treatment with saikosaponin (SSa) [108]. Through a high dose of AU, GLT-1 protein levels have been shown to be upregulated with GABA levels as well as the GABA_A_ receptor subunit a1 (GABA_A_Ra1) [111].

Another reason for changes in neurotransmitter levels is the stimulation of cytokines in the brain [57]. They are mostly released from activated microglia in pathological conditions but could enable glial cells to release glutamate in a calcium-dependent manner [58,156]. In addition, proinflammatory cytokines, such as TNF-α and IL-1β, inhibit the ability of astrocytes to take up extracellular glutamate by reducing the mRNA level of glutamate transporters and also stimulating glia to release glutamate to the extracellular space [48,96]. The gap junction is one of the crucial proteins in astrocytes for communication and forming the astrocytic syncytium. In a kainic acid (KA)-induced epilepsy model, astrocytic connexins were shown to aggravate epileptic signals [118]. In addition, inflammatory cytokines inhibit astrocyte gap junction coupling in cell culture experiments. Following treatment with levetiracetam in LPS-induced gap junction coupling-inhibited mice, the uncoupling effects of LPS were entirely prevented [146]. Astrocytes and microglia are affected by cytokines and release glutamate from the microglia through the connexin 32 hemichannels as well as glutamate transporters [119]. Increased extracellular glutamate levels are thought to be the cause of epilepsy. This is shown by the conventionally used epileptic drugs. Carbamazepine decreases the astrocytic release of glutamate, which is increased in association with chronic cytokine release [120]. By increasing the expression of EAAT2, a glial cell glutamate transporter, pilocarpine-induced epilepsy can be relieved [121]. Blocking the astrocytic cannabinoid 1 (CB1) receptors have been shown to decrease the duration of epilepsy, even though the onset was unchanged [122]. In astrocytes, by increasing the astrocytic GLT-1, the extracellular glutamate level decreases, and these changes reduce the seizures in tuberous sclerosis (TSC)-conditioned KO mouse model [92]. In addition, the treatment of AU also attenuates the activation of glial cells [111]. It has long been known that excessive amounts of glutamate in the extracellular space can kill neurons [59]. The concentration of glutamate is maintained within the normal range through the uptake function of astrocytes and the adaptation range of glutamate release from the glial cells [60,61].

## 4. Conclusions

**Figure 3:**
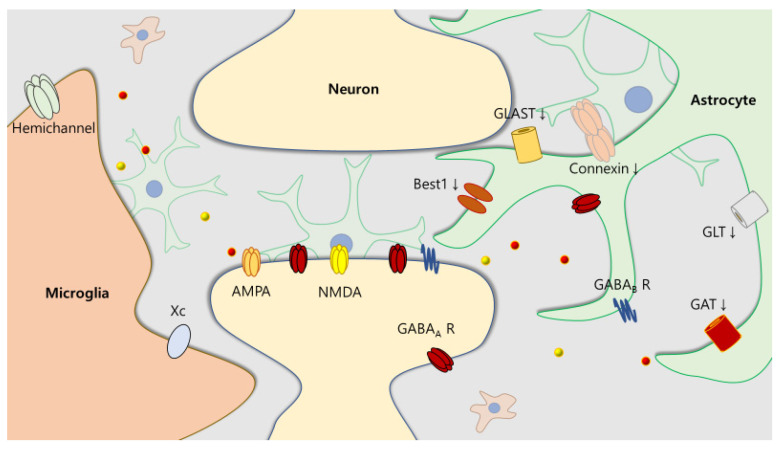
Alteration of transporters and channels in neurodevelopmental disorders. Several transporters and channels on glial cells are responsible for the presence of a pathological state. In the epileptic brain, the expression of glutamate transporter (GLT), glutamate aspartate transporter (GLAST), GABA transporter (GAT) [79], and bestrophin-1 channel (Best1) [12] on astrocytes are decreased (↓). Changes in GABA receptors have been reported, and neurotransmitters such as glutamate and GABA are diffused by gap junction hemichannels composed of connexins [145] or microglial hemichannels [119].

Our review highlights the presence of various glial interactions with neurons in several neurodevelopmental disorders. Some mechanisms, such as neuroinflammation, imbalance of excitation and inhibition, and neurotransmitters, are shared in ASD, ADHD, and epilepsy. Both patient- and animal model-based neurodevelopmental disorders demonstrate an increased level of cytokines in the brain. In addition, the alteration of neurotransmitters creates an imbalance relative to the normal status. This imbalance could be induced by changes in the expression levels of receptors and transporters, the modification of released gliotransmitters, and through dysfunction of uptake (Figure 3).

Even with the high comorbidity of neurodevelopmental disorders, studies on the common mechanisms underlying ASD, ADHD, and epilepsy are rare. In addition, as the functions of neurons have been studied extensively previously, it is important to study the role of glial cells in connection with neurons. As therapeutics for neurodevelopmental disorders do not target the exact mechanisms of the disease, the drug resistance rate is relatively high. For example, there are no prominent therapies for the symptomatic relief of ASD. Methylphenidate, a drug commonly used for the treatment of ADHD, can cause hyperactivity, which is also a symptom of ADHD [104]. In the case of epilepsy, many types of anticonvulsants have been developed and are in clinical use. However, valproic acid is a commonly used anticonvulsant that can cause ASD in children [71]. These side effects caused by medications are due to the lack of understanding of the complex neuron-glia interactions. Concerning the development of new therapeutic targets, the common pathways in neurodevelopmental disorders give us hope that more feasible therapies with fewer side-effects can be developed for growing children.

In this review, we did not discuss the notable sex ratios of neurodevelopmental disorders. However, sex is one of the most important factors associated with this topic and is prominent in ASD and ADHD [123,129]. The different sex rates of children suffering from a disease are important to study the mechanisms and susceptibility of that disease. It is also important to recognize that neurodevelopmental disorders are accompanied by the behavioral and social effects of the medication. The response to the disease differs depending on the sex of the child, and doctors and parents need to tailor their recommendations accordingly [172]. Researchers suggest considering the sex effect not only from a sociocultural perspective but also in terms of biological effects [62,147].

The degree of sex imbalance is more prominent in ASD, but it also exists in ADHD [173]. Depending on the sex, the subtypes of ADHD are different, even if the severity of the disorder is similar [173,174]. ADHD is often reported to cause different problems that are sex-dependent, and the effect of ADHD on academic performance is also noted to differ by sex [175]. Some have reported that, even though the prevalence of ADHD varies by sex, specific alterations are not found according to sex, but instead that it should be considered during the diagnosis [176].

Finally, the effect of puberty should be considered in patients with neurodevelopmental disorders. Even though ADHD symptoms in adults are still controversial, the adult ADHD onset takes place during early childhood and is merely a continuation of those early onset symptoms [180]. Adults with ADHD show significant inattention as well as hyperactivity, and increased exposure to drug abuse [148,177]. The main theory behind neurodevelopmental disorders, including ADHD, is that they are not relieved over time, but instead that patients learn to manage the symptoms better [178]. Further studies in adults with ADHD are required to differentiate the characteristics of children with ADHD and adult ADHD, and determine the effect of time on neurodevelopmental disorders.

## Figures and Tables

**Table 1:** Classification of the types and methodological components of papers contributing to the review.

Types of Paper					
Review	Research				Others
Systematic	Animal		Human		Mathematical
[3,7,14,19,21,22,23,24,25,26,27,28,29,30,31,32,33,34,35,36,37,38,39,40,41,42,43,44,45,46,47,48,49,50,51,52,53,54,55,56,57,58,59,60,61,62]	Genetic	[1,2,4,5,8,9,10,11,12,17,18,63,64,65,66,67,68,69,70,71,72,73,74,75,76,77,78,79,80,81,82,83,84,85,86,87,88,89,90,91,92]	Case	[13,15,16,93,94,95,96]	[97,98]
Meta-analysis	Pharmacological	[6,11,12,18,20,63,77,99,100,101,102,103,104,105,106,107,108,109,110,111,112,113,114,115,116,117,118,119,120,121,122]	Cohort	[123,124,125,126,127,128,129,130,131,132,133,134,135,136,137,138,139,140,141,142,143,144,145,146,147,148]	
[149,150]	Environmental	[16,71,151,152,153,154,155,156]	Comparative	[157,158,159,160,161,162,163,164,165,166,167,168,169,170,171,172,173,174,175,176,177,178]	

**Table 2:** ASD/ADHD models with observed neuron-glia interactions. ASD: autism spectrum disorder; ADHD: attention-deficit/hyperactivity disorder.

ASD	Model
Genetic	Shank2 KO [24], Shank3 KO [70], NLGN3 R451C KI [67], NLXN1 KO [24], TSC1 HT [64], BTBR [70], MeCP2 mutant [65], Scn1a HT [64], PTEN mutant [158]
Pharmacological	Valproic acid (VPA) [71]
Environmental	Methyl mercury [127], maternal immune activation (MIA) [99,100,126], polyinosinic:polycytidylic acid (poly I:C) [63]
**ADHD**	**Model**
Genetic	Dopamine transporter (DAT) mutant [33], NK1R-KO [33], SNAP25 mutant [33], Git1 KO [160,161], Cdk5 KO [72], nicotinic acetylcholine receptor (nAChR) ß2-KO [33]
Pharmacological	Ethanol [162], methylazoxymethanol [104,105]
Environmental	Neonatal X-radiation, hypoxia, heavy metal exposure (lead, cadmium), oncogenic environmental exposure (polychlorinated biphenyl (PCB)) [33]

NLGN: neuroligin; NLXN: neurexin; TSC: tuberous sclerosis; MeCP2, methyl CpG binding protein 2; Scn1: sodium channel protein type 1; PTEN: phosphatase and tensin homolog; NK1: neurokinin; SNAP: synaptosome associated protein.

**Table 3:** Epilepsy models with observed neuron-glia interactions.

Epilepsy	Model
Genetic	AP (Activator Protein)-1 KO [108], SCN1A KO, DBA/2 KO [109] **Lafora disease:** Malin KO, Epm2a, Epm2b [89,90]
Pharmacological *	Pentylenetetrazol (PTZ), pilocarpine (PA), kainic acid (KA)
Environmental	Electrical stimulation, brain injury

* To study epilepsy, pharmacological models are commonly and widely used.
